# CNN based method for classifying cervical cancer cells in pap smear images

**DOI:** 10.1038/s41598-025-10009-x

**Published:** 2025-07-04

**Authors:** Remita Austin, R. Parvathi

**Affiliations:** School of Computer Science and Engineering, Vellore Institute of Technology, Chennai, 600127 India

**Keywords:** Classification and taxonomy, Computational models, Computational platforms and environments, Data integration, Data mining, Data processing, Image processing, Machine learning

## Abstract

The absence of reliable early treatment serves as one of the main causes of cervical cancer. Hence, it is crucial to detect cervical cancer early. The biggest challenge in diagnosing cervical cancer early is that it is asymptomatic until it develops into invasive carcinoma. In medical applications, the use of machine learning and deep learning is successful as a classifier in the preliminary identification of cancerous cells in the cervical region. In our study, we present a CNN-based method for the classification of cervical cancer cells. We present a method for accurately classifying Pap smear images into abnormal or healthy cells by extracting essential information using a variety of deep-learning approaches. Experiments are performed using the SIPaKMeD and Herlev datasets. Several pre-trained convolutional neural network (CNN) models are used via transfer learning methods, hence predicting and evaluating the accurate classifier with the best optimal solution. Classification of cervical cell clusters in whole slide images (WSI) has usually comprised two stages: segmentation to extract individual cell patches, and subsequently single-cell categorization. As a result, segmentation accuracy determines the classification pipeline’s performance. We propose a direct classification of WSI cervical cell groups without segmentation and demonstrate that segmentation is not strictly necessary for good classification results. Our solution outperformed prior methods and benchmarks, with an accuracy of 96.74% for WSI patches and 97.55% for full-cell images for the SIPaKMeD dataset, and an accuracy of 90.42% for the Herlev dataset. The results show that the suggested approach may accurately distinguish cervical cancerous and non-cancerous cells.

## Introduction

Cervical cancer is characterized by the unusual spread of cell growth. Malignant cells have the ability to spread to neighboring tissues and organs. Cervical cancer develops at the bottom of the uterus in the cervical area. Abnormal uterine cells multiply and grow in aberrant cell cycles. Women with early-stage cervical cancer have no symptoms since it is a slow-growing malignancy that does not spread to other organs of the body. Early identification of the disease is totally treatable and preventive due to the disease’s prolonged pre-malignant phase^1^.

For women between the ages of 15 and 44 in India, this condition is the most prevalent kind of cancer^1^. Cervical cancer is the second most prevalent type of cancer among females, according to GLOBOCAN 2020. The incidence of cervical cancer has decreased from 17% to 9%. This represents a significant decrease in cervical carcinoma incidences among Indian women due to early diagnosis and treatment^2^.

In 99% of cases, human papillomaviruses (HPV) are the main cause of cervical cancer. It develops in women who have persistent HPV infections. The cancer nodules can transfer infections to other parts of the body and cause serious medical problems. Studies show that if precancerous lesions are identified early during cytological screening and HPV tests, cervical cancer can be treated. The current HPV prevention methods include vaccine, detection, and treatment. By employing these prevention methods, cervical cancer can be eliminated^3^.

Apart from HPV, suffering from human immunodeficiency virus (HIV) or another illness that makes it challenging for the immune system to combat illness can raise the likelihood of cervical cancer. Cervical cancer strikes HIV-positive women six times more often than HIV-negative women. Then having multiple sexual partners raises the risk of developing cervical cancer. Another possibility is smoking. Tobacco harmful byproducts have been detected in smokers’ cervical mucus. According to studies, these compounds trigger damage to DNA in cervical cells, which may contribute to cervical cancer growth. Tobacco usage also reduces the ability of the immune system to fight infections caused by HPV. Then, long-term use of contraceptive pills, for five or more years, raises the risk^4^. Aside from these factors, having three or more children raises the likelihood of cervical cancer. This is assumed to be because having sexual relations increases the likelihood of infection. Furthermore, fluctuations in hormones throughout pregnancy may render women more susceptible to contracting HIV or the growth of cancer. According to certain research, women who have had infections caused by chlamydia in the past or are currently infected have an increased chance of contracting cervical cancer. Certain studies suggest that Chlamydia bacteria helps in the development and survival of HPV in the cervix, increasing the risk of cervical cancer.

The Papanicolau Smear test is one among many widely used traditional screening assessments for detecting cervix cell changes. The screening procedure aids in the detection of abnormalities in their early precancerous phase. In more developed nations, comprehensive and mandatory cervical cancer screening has helped to reduce the disease’s incidence and death rates. However, in developing nations, a lack of amenities, inadequate information, and the effects of the disease raises the overall prevalence and mortality of cancer, making it a severe concern to women^5^. An experienced pathologist detects cancer by manually analysing the anatomical features of cells in microscopic specimens. Because this is dependent on the expertise and knowledge of the professional, it could result in erroneous outcomes. Automated systems and medical image processing are used in the analysis of cancerous cells. As new procedures are invented, they become more economical and less labor intensive^6^.

In recent years, the application of machine learning approaches in biomedicine has pushed the development of prediction models to identify various diseases. Deep learning approaches have expanded rapidly, and many scientists have emphasized their importance in scientific studies for cancer diagnostic prediction. State-of-the-art machine learning (ML) techniques have made it possible to create an entire system for healthcare diagnosis that can function reliably, in actual time, and without the need for human intervention^7^. However, certain automated screening tools for Pap smears have drawbacks, such as reduced sensitivity, doubts about their cost-effectiveness, and an inability to identify cases of early abnormalities.

The current medical and disease condition, in which there is a shortage of skilled medical professionals and a high incidence and death of cervical cancer, served as the inspiration for the study. The fact that proactive disease prevention and early detection measures can save lives is also a major motivator for this project. This research is motivated by deep learning solutions in biomedical imaging, which are critical for early detection. As previously stated, this study contributes to reducing the shortcomings of manual analysis that may result in an incorrect diagnosis. It can also be of great assistance to doctors. Furthermore, a prompt and accurate diagnosis is a crucial requirement before continuing treatment. Therefore, as the number of patients is increasing day by day and given the incidence of the disease, it is significant to use deep learning methods in the detection of cervical cancer.

The proposed study uses SIPaKMeD and Herlev datasets since these are the most recent cervical cell image datasets and consist of pap smear test images with single-cell and whole slide images required for determining as normal or abnormal cells.

The main objectives of the proposed cervical cancer diagnosis system are as follows. First, the system aims to enhance the efficiency of image processing and improve visualization by accurately identifying anomalous patterns in Pap smear images. To prevent overfitting, appropriate techniques will be applied during training. The study also analyses classification performance on whole slide image (WSI) data, both with and without segmentation strategies using transfer learning. Furthermore, it investigates the functions that significantly influence network accuracy using the SIPaKMeD and Herlev datasets. A custom deep neural network will be designed and trained to distinguish between cancerous and non-cancerous images across various cervical cell types, thereby facilitating early diagnosis of cervical cancer. Finally, the study seeks to develop the most effective classifiers for reliable cervical cancer diagnosis.

## Related work

The work in^8^ implemented support vector machines on the Herlev pap-smear image dataset. To distinguish the cells belonging to each one of the Herlev classifications, despite the fact that some of them had been mixed, the authors have taken characteristics from both the nucleus areas and the entire cell. These two approaches use principal component analysis, also known as PCA, to lower the overall dimension within the feature vector to deal with the multiplicity of features computed. Here, active contour models have been applied for segmentation. The model accuracy was 95%.

In^9^, a deep learning method using DenseNet-201 was employed for feature extraction to detect ovarian cancer in histopathology images. The PLCO dataset was used in the characterization cycle and achieved the highest accuracy of 94.73%, exactness of 0.91, review score of 0.90, and f1-score of 0.95.

The authors reported a cervical cancer detection system with transfer learning for early diagnosis^10^. Before the deep learning model was trained, pap smear images were processed with a median filter-based preprocessing method to eliminate noise and improve the classification. SqueezeNet, AlexNet, ResNet-50, VGG-19, and InceptionV3 are five well-known pre-trained networks that have been used and compared for this problem. SqueezeNet outperformed other neural structures in terms of validation accuracy, achieving a score of 96.90%. With this method, cervical cancer could be diagnosed in a confidential, affordable, and quick manner.

The review in^5^, provided a summary of the cutting-edge approaches described in the prominent literature on automated diagnostic tools for cancer diagnosis. This study highlights some of the shortcomings and flaws in the methodologies examined and provides information to help evaluate the methodology employed in the literature. The study highlighted the potential for developing an automated, cost-effective method for classifying diseases, which should be very helpful for nations with few resources and treatment options.

In^6^, a novel approach was suggested that makes use of transfer learning and technology for progressive scaling. The evaluation made use of the SIPaKMeD dataset. By progressively elevating the resolution of the image from 224x224 pixels to 256x256, 512x512, and 1024x1024 pixels, the model was iteratively trained using this strategy. The WSI image multi-classification had an accuracy rate of up to 99.70%.

A recent study by^11^ suggested transfer learning-based approaches to aid in the categorization of cervical cancerous cells. They investigated six different approaches for determining the types of cervical cells which included three existing models as features, shallow CNN, which had just two layers of convolution and two layers for max-pooling, VGG-16, and CaffeNet as a feature extraction method, and two classification algorithms, extreme learning machine and autoencoder. For system testing and training, they made use of the Herlev dataset. The recommended hybrid CNN-ELM-based model obtained 99.50% and 91.20% accuracy on 2-way and 7-way categorization, respectively. However they’ve also recommended incorporating hand-crafted features into their system to enhance the results furthermore.

Data augmentation approaches were utilized by^12^ to reduce overfitting and compensate for the relatively small databases used in the automated identification of cervical carcinoma. The authors used statistics from the National Cancer Institute’s database to determine if a cervix picture was normal/CIN 1 or CIN2+. Based on an idea of layer concatenation, a ColpoNet CNN algorithm was developed. In comparison to previous CNN designs, a precision of 81.35% was achieved. According to the authors, 3000 epochs were used to achieve an accuracy of 83.95%.

Using deep learning and transfer learning approaches,^13^ created cervical cancer predicting model to categorize images of the cervix into Type 1, Type 2, or Type 3 classes. ConvNet was built using the three relevant models, InceptionV3, ResNet50, and VGG19, for categorizing the images of the cervical region. The InceptionV3 model outperforms ResNet50 and VGG19 models with an accuracy of 96.1% according to the results.

According to^14^, their research offers a low-cost diagnostic system that makes use of an algorithm for analysing images of cervical tissue stained with hexatin and eosin. In this investigation, the cervical tissue was photographed using a smartphone put on the head of a light microscope, however, the magnification could not be recorded. Four different types of multiple-instance learning algorithms with various instance sizes had their classification performance objectively tested. The classification tasks are characterized as deep multiple-instance learning problems.

The study in^15^ combined two deep learning algorithms to accurately recognise and classify cell clusters using the Bethesda system. Without any observable false negatives, the cell identification utilizing YOLOv4 managed to identify every cell with abnormalities ahead of ASC-US. The ResNeSt algorithm was used to classify the observed cell pictures, having 90.5% average accuracy and 70.5% F-measure score.

In^16^, an AlexNet-based deep convolutional neural network model was trained with three-fold cross-validation. During the training and testing processes, simultaneously the original RGB and the enhanced images were employed. In this study, it was discovered that increasing the number of training samples using image processing resulted in a 3.85% improvement in model accuracy. They achieved recognition accuracy of 93.33% for the original image and 89.48% for the augmented image, respectively.

A novel CervixNet model has been introduced by^17^ and advances image improvement on cervigrams as well as segmenting and categorizing the region of interest (ROI) to provide better treatment. TFor the categorization, they used a neural network based on the Hierarchical Convolutional Mixture of Expert and Mask RCNN for ROI extraction. Small datasets are a fundamental issue in biomedical imaging, and HCME was used to address the overfitting concerns. The accuracy of this approach was 96.77%.

The study in^18^ employed a combined approach of three deep neural network architectures, including Deep SVDD, RetinaNet, and a custom CNN model to detect the cervix on smartphone-captured photos. The results of the study demonstrated that the ensemble approach outperformed separate deep CNNs and had an average accuracy of 91.6% and an F1-score of 0.890.

The work done in^19^ suggested a deep learning (DL) architecture for whole-slide cervical cancer detection. They devised a tier three categorization scheme: Initially, a CNN scans an image with a low resolution of the entire slide for abnormal areas. Following that, another CNN analyses images with high resolution of the points of interest suggested by the previous CNN and returns a likelihood that the area in question includes a lesion cell. At last, an RNN analyses the top ten sections to generate a total score for the entire slide.

In^20^, three models were used based on transfer learning to develop data-specific features for cervical cancer diagnosis in pap smear images: MobileNetV2, InceptionV3, and Inception-ResNetV2, all comprising extra layers. They suggested a clustering strategy based on three different distance measurement techniques to reduce the discrepancy between the expected and actual values. While MobileNetV2, InceptionV3, and Inception-ResNetV2 were each executed independently, it obtained 95.30%, 93.92%, and 96.44%, respectively. The performance improves to 96.96% after using the suggested ensemble strategy, outperforming the individual models.

According to the study in^21^, a 2D slice of CT scans was used to describe a deep convolutional neural network for automatically segmenting the organs subjected to risk in high-dose frequency brachytherapy of cervical carcinoma. In the suggested models, lengthy and shorter connections with skips were used, ResU-Net and U-Net, to increase the accuracy of feature extraction and segmentation. This study gathered images from 113 patients with extremely advanced cancer of the cervix. The review employed established quantitative measures such as the DSC, ASSD, and HD.

A cervical cancer diagnosis was presented by^22^ utilizing a colposcopy image and an ensemble deep-learning network. In this study, the cervix was identified using colposcopy images and classified using a DCNN. Two models-VGG19 and CYENET-were then suggested as a result of this technique. The study’s findings demonstrated that the proposed CYENET model had a high level of 92.3% accuracy in classifying abnormal cells.

The study in^23^ shows how a deep learning model may be used to categorize cervical cancer cells. It uses four dataset-based categorization models. Model A is a 10-layer, straightforward CNN. Model A is equipped with the Model B Spatial pyramid pooling layer before the FC layers (CNN+SPP). In this study, cervical cells are automatically classified using first-time inception and the SPP layer. Inception and SPP are used in Model D to create a flexible model with great accuracy. Comparing the effectiveness of these models yielded the top model, with an AUC value of 0.997. One of the open problems that was left is the limited volume of data. As a result the authors suggested the use of image generation technology to improve the model performance in future.

A Computer-Aided Diagnosis System was proposed in^24^ to aid healthcare professionals to identify cervical cancer. It comprises of two processes: preliminary processing and categorization. Greyscale, histogram equalization, and median filtering are utilized as preprocessing. The Deep Belief Network approach is used for data classification. The advantages of the DBN approach for the identification of cervical cancer produced the best accuracy results of 84%. This study proves to be useful for identifying the features, but it detects only fewer data samples.

The work done in^25^, presented a system using deep learning for identifying the types of cervical cells. They used hybridized deep fusion approaches to combine different deep learning techniques to capture as much information as possible to improve the accuracy of classification. Using cervical Pap smear images from the SIPaKMeD dataset, this strategy is evaluated by comparing the outcomes of DL models with the later fusion method.

The classification systems based on k-NNs and ANNs were suggested in^26^. The tests made use of the Herlev database. The k-NNs approach had an accuracy of 88%, whereas the ANNs method had an accuracy of 54%. The study in^27^ employed 20 shape and position features as well as other machine learning approaches. The stated accuracies for the 2-class problem ranged between 94% and 97% depending on the strategies used, and 72% and 80% for the 7-class problem.

In^28^ a pre-trained and customized CNN architecture was built on GoogleNet, AlexNet, ResNet, and DenseNet which is utilized to categorize cervical cancer cells, where both nucleus and cytoplasm segmentation is necessary. The study in^29^ compares the classification performance of five deep learning models namely AlexNet, ResNet-101, VGG-19, DenseNet-161, and SqueezeNet, on the cervical cancer database, with DenseNet-161 offering the greatest accuracy.

Recent studies in automated cervical cancer screening using deep learning as shown in^30^ have made notable advances. The author conducted a comprehensive evaluation of 13 pre-trained CNN architectures including DenseNet-201, ResNet-50, VGG-16, and Xception-for 7-class classification on the Herlev dataset. DenseNet-201 achieved the highest accuracy, and the study explicitly avoided any segmentation or hand-crafted feature extraction, relying on transfer learning alone. This contrasts with earlier methods that integrated pre-processing pipelines, suggesting a shift toward simpler, more generalizable architectures.

The study in^31^ introduced a novel Transformer-based method using cross-attention and a compact latent Transformer module for cell-level classification. It achieved 93.7% and 94.6% accuracy on the SIPaKMeD (3-class) and Herlev (2-class) datasets, respectively. While it did not outperform top CNNs on the Herlev dataset, it demonstrated strong performance without explicit segmentation, signifying potential for future attention-based architectures.

**Table 1:** Summary of state-of-the-art methods for cervical cancer detection.

Study	Method	Dataset	Accuracy (in %)	Strengths	Weaknesses
Bhatt et al. (2021)	Progressive scaling with transfer learning	SIPaKMeD	99.7	High-resolution iterative training	High computational cost
Ghoneim et al. (2020)	Hybrid CNN + ELM	Herlev	99.5 (2-class), 91.2 (7-class)	Combines CNN with advanced classifier	Needs hand-crafted features
Csenturk et al. (2022)	Transfer learning (SqueezeNet, etc.)	Pap smear images	96.9	Compared multiple DL models; fast, cost-effective	Performance varies across models
Pramanik et al. (2022)	Ensemble (MobileNetV2, InceptionV3, Inception-ResNetV2)	Pap smear images	96.96	Ensemble outperforms individual models	Complex model coordination
Gorantla et al. (2019)	CervixNet (HCME + Mask RCNN)	Cervigrams	96.77	Custom segmentation + classification pipeline	Small dataset constraint
Dhawan et al. (2021)	Transfer learning (InceptionV3> ResNet50, VGG19)	Cervix images	96.1	Strong performance across models	Dependent on pre-trained networks
Wadhwa et al. (2021)	DenseNet-201	PLCO	94.73	Deep feature extraction	Slightly below top-tier accuracy
Deo et al. (2024)	CerviFormer (transformer)	SIPaKMeD, Herlev	93.70 (SIPaKMeD), 94.6 (Herlev)	Novel architecture, uses attention mechanisms	Slightly lower accuracy, no image enhancement

From the literature review, we saw that few studies posed issues with respect to detecting the cells accurately. Then some others had issues with the dataset they used since those were of low image quality and had a small data volume. A few studies didn’t perform any preprocessing techniques over the image data, which would have increased the model accuracy. Some others didn’t apply data augmentation, which also would help to achieve better accuracy. Then the accuracy could have been higher for a few studies by applying better classifiers for the classification of cervical cancer cells. Table 1 summarizes a selection of state-of-the-art approaches that have addressed many of these challenges and achieved high accuracy through the use of deep learning architectures, ensemble techniques, preprocessing, and high-resolution imaging.

## Material and methods

### SIPaKMeD dataset

The most latest accessible cervical image dataset is SIPaKMeD. The initials SI (Superficial/Intermediate), P (Parabasal), K (Koilocytotic), M (Metaplastic), and D (Dyskeratotic) are derived from the names of the various cell types in the data collection. It was made available in 2018 to aid in the early diagnosis of cervical cancer. 4049 single-cell images were cropped by^32^ from 966 cervical tissue images taken from Pap smear slides.

**Fig. 1:**
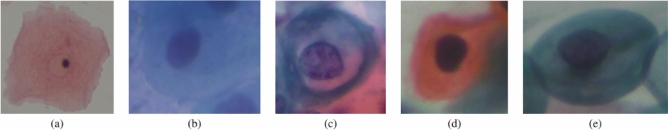
Full cell images of five classes: (**a**) superficial-intermediate, (**b**) parabasal, (**c**) koilocytotic, (**d**) dyskeratotic, (**e**) metaplastic^32^.

In this dataset, the images of five cell types were included. Figure 1 displays sample images provided in the dataset. Cell types are categorized according to their location and degree of maturity. Superficial/intermediate and parabasal cells are normal or non-cancerous cells. Metaplastic cells are benign but suspicious precancerous lesions, meaning they indicate a high likelihood of developing cancer, while koilocytotic and dyskeratotic cells are abnormal cells. 1618 of the images are in a normal class, and 2449 of them are abnormal cells. In this study, we will classify cervical cells as normal or abnormal to distinguish between early-stage cancer and non-cancerous cells.

**Fig. 2:**
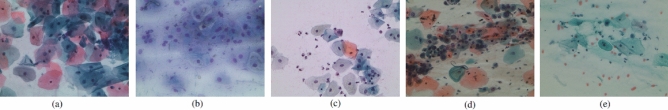
Whole slide images of five classes: (**a**) superficial-intermediate, (**b**) parabasal, (**c**) koilocytotic, (**d**) dyskeratotic, (**e**) metaplastic^32^.

Apart from the single-cell images, this dataset also consists of whole slide images. It consists of over 966 cluster cell images of Pap smear slides with five different cell types. The cell clusters (WSI patches) that belong to the various classes are shown in Fig. 2. Here 126 images belong to Superficial-Intermediate, 108 images belong to Parabasal, 238 images belong to Koilocytotic, 271 images belong to Dyskeratotic, and 223 images belong to the Metaplastic class.

### Herlev dataset

**Fig. 3:**
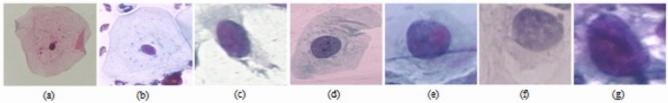
Single cell images of seven classes: (**a**) superficial squamous epithelia, (**b**) intermediate squamous epithelia, (**c**) columnar epithelial, (**d**) mild squamous non-keratinizing dysplasia, (**e**) moderate squamous non-keratinizing dysplasia, (**f**) severe squamous non-keratinizing dysplasia, (**g**) squamous cell carcinoma in situ^33^.

The Herlev dataset was developed utilizing an electronic camera microscope at the Herlev University Hospital, Denmark, and comprises individual cell images with nuclear locations. There are a total of 917 cells with seven different classes. There are three normal classes and four abnormal classes. Figure 3 displays the Herlev dataset sample pap test images.

Skilled pyrotechnicians and medical professionals classified each cell as one of seven types: superficial squamous epithelia, intermediate squamous epithelia, columnar epithelial, mild squamous non-keratinizing dysplasia, moderate squamous non-keratinizing dysplasia, severe squamous non-keratinizing dysplasia, and squamous cell carcinoma. The cells can be distinguished from one another by their morphological characteristics, such as the shape of the cell, nucleus dimensions, nuclei-to-cytoplasm proportion, nucleus opacities, nucleus dying magnitudes, cytoplasm opacities, and cytoplasm dying magnitudes. There are 242 images in the normal class and 675 images in the abnormal cell class.

### Tools used

The research study was conducted in the Google Colab environment using Python 3.10. A GPU-enabled runtime with an NVIDIA Tesla K80 and 12GB RAM was employed to accelerate computation, particularly for feature extraction using pre-trained models. The cv2 module from the OpenCV library (version 4.7.0) was used for general image processing tasks such as loading, resizing, and blurring images. TensorFlow’s Keras API (TensorFlow version 2.12.0) was utilized to load pre-trained convolutional neural network models and extract feature vectors from input images. The Matplotlib library (version 3.7.1) was used to visualize dataset images and generate plots, including accuracy and performance charts. The NumPy library (version 1.22.4) supported efficient numerical computations and was used to convert image files into arrays and split the dataset into training, validation, and testing subsets. The Pandas library (version 1.5.3) was employed to organise classification metrics and store model performance results in structured DataFrame formats.

### Design of proposed system

**Fig. 4:**
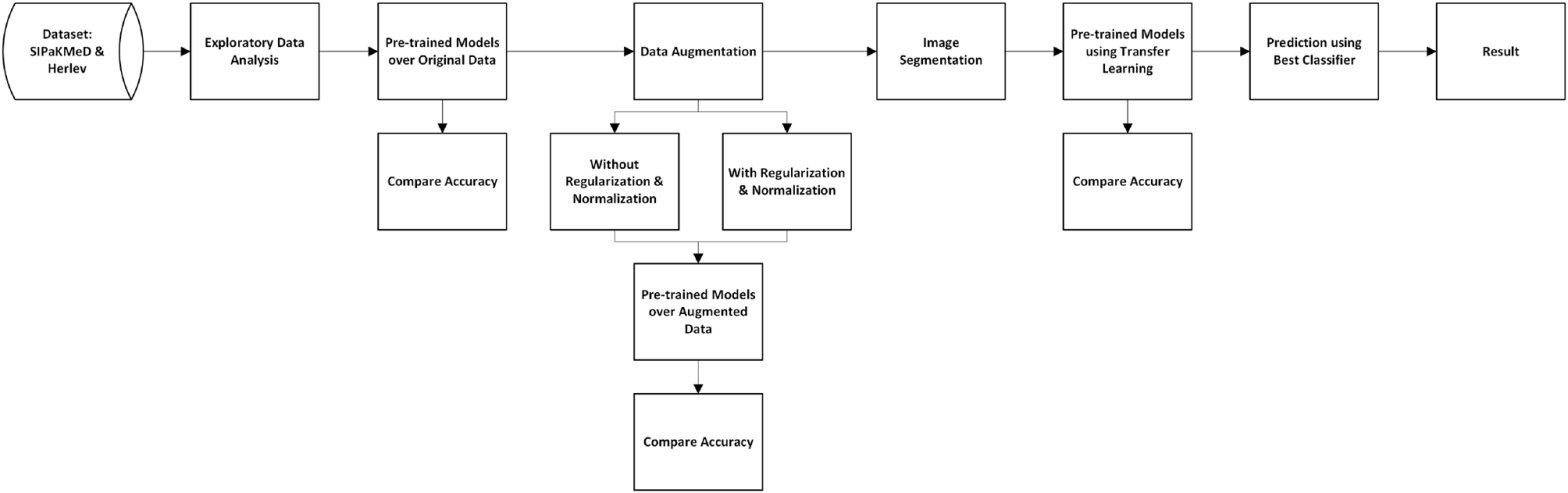
A block diagram of the proposed methodology.

The proposed cervical cancer diagnosis system has a series of six steps involving data exploration, applying pre-trained models over the original dataset, data augmentation for increased input data size, applying pre-trained models over the augmented data, image segmentation, and applying pre-trained models using transfer learning methods that give better accuracy. In the first step, we have the data collection of pap smear test images, followed by the exploratory analysis of the images. Then we apply pre-trained models over the original data and observe the accuracy. Then we apply data augmentation methods with and without regularization and normalization methods and find the accuracy of the CNN models over the data. Then, image segmentation techniques are employed, and we implement the models using transfer learning over the segmented images. Finally, we classify the images as abnormal or normal cells and compare and evaluate the results. Fig. 4 depicts the proposed system’s block diagram.

### Data augmentation

To avoid overfitting, data augmentation is used to enhance the cardinality of the training dataset. It contributes to the overall accuracy of the convolutional layer structure’s network. Thus, data augmentation techniques such as rotation, flipping, shifting, and zooming were used to increase the total number of images. When training is performed with moderate training data sizes, the outcomes are poor.

In the case of small data sets, standard machine-learning techniques can outperform other deep-learning approaches in terms of accuracy and results. As a result, data augmentation is performed to improve the training dataset’s cardinality while avoiding overfitting. The images are generated automatically by performing multiple minor image changes on the region of interest, which boosts the deep-learning models’ learning capabilities.

Data augmentation is critical for preprocessing the image to increase the number of images in the data while preventing overfitting during training. The following are the key parameters for augmentation:

- Rescaling: It is set to 1./255 i.e., it divides each pixel value by 255.
- Rotation range: For arbitrary rotations, the range is integer degrees. It has a value of 40$^{\circ }$.
- Width shift range: Its value may contain a float, for example, a 1D array as well as an integer, thus it is set to 0.2 for this study.
- Height shift range: It can have a float value, similar to a 1D array as well as an integer. It has a value of 0.2.
- Shear range: It is a shear magnitude, which is represented by degrees as a shear angle in the anticlockwise direction. It has a value of 0.2$^{\circ }$.
- Zoom range: Zoom range: It is a range that has been defined for arbitrary zoom. Its value is defined as a float value, then zoom will take place in the range [1-zoom_range, 1+zoom_range]. It is set to 0.2 for the purpose of this study.
- Horizontal flip: It randomly flips the input horizontally. It is of the Boolean type, and it is set to True for the current analysis.
- Vertical flip: It randomly flips the input vertically. It is of the Boolean type, and it is set to True for this research.
- Fill mode: It specifies how points outside of the input boundaries are filled. It can have a value of nearest, constant, reflect, or wrap. It was chosen as the ’nearest’ for the purpose of this study.
- Blur: For blurring the image, we have used the blur() method in OpenCV which takes the image and kernel size as parameters. The image becomes blurrier as the kernel size increases.

### Regularization and normalization

Regularization parameter is used to reduce overfitting. L2 regularization techniques are classified as weight/parameter regularization. This type of regularization keeps the weights of the neural network small by adding a penalizing term to the loss function. Setting the regularization parameter to zero may reduce the generalizing capability of the network. Its value is mostly on a logarithmic scale between 0 and 0.1. It adds some penalties to the weights. We use the kernel_regularizer argument to apply regularization only to the weights of the network. The L2 Regularization parameter was set as 0.001 in our study.

Along with the regularization method over the augmented data, the batch normalization technique was introduced between the layers of pre-trained models. It refers to a normalization approach used between each layer of a deep neural network instead of relying on raw input. Rather than processing the complete data set, it is processed in mini-batches. It facilitates learning by speeding up training and utilizing greater learning rates. The following process takes place in the batch normalization layer:

1. The mean and variance of the input layers are computed.
  1 $$\begin{aligned} Batch\,Mean,\, \mu _B = \frac{1}{m} \sum _{i=1}^{m} x_i \end{aligned}$$
  2 $$\begin{aligned} Batch\,Variance,\, {\sigma _B}^2 = \frac{1}{m} \sum _{i=1}^{m} (x_i - \mu _B)^2 \end{aligned}$$
2. The layer inputs are normalized using the previous batch statistics that were computed.
  3 $$\begin{aligned} \overline{x_i} = \frac{x_i - \mu _B}{\sqrt{{\sigma _B}^2 + \epsilon }} \end{aligned}$$
3. Finally, shifting and scaling of the normalized input take place to obtain the output.
  4 $$\begin{aligned} y_i = \gamma \overline{x_i} + \beta \end{aligned}$$
  where $\gamma$ and $\beta$ are learned while training in combination with the network’s original parameters.

### Pre-trained CNN models

The most extensively utilized deep learning network for image categorization is the ConvNet or the convolutional neural network. The ConvNet is a network of neurons with three layers: input, hidden, and output. Because of the thousands of layers that are hidden, it is a more complex neural network. The input layer receives the unprocessed values of pixels from the image, while the output layer consists of neurons matching the number of output classes. The final layer of the convolution is an entirely interconnected layer that utilizes the activation layer SoftMax. Our proposed system employs seven pre-trained models, which are stated below:

- ResNet-50: ResNet-50 is built on the residual learning architecture to make training a deeper network easier. This model seeks to prevent inaccurate results as the model gets deeper. It tackles the vanishing gradient problem and accelerates the training speed.
- VGG-16: It is a CNN that has 16 layers deeper. It focuses on creating deeper networks to improve classification accuracy. Although it takes a while to train compared to other models, this model is straightforward to understand.
- VGG-19: It is another prominent deep-learning model used for image classification and is named after the Visual Geometry Group. It comes in a variety of variations such as VGG-16, VGG-19, and so on. VGG-19 has 19 layers: convolution layers make up 6, max pool layers are 5, 3 completely linked layers and SoftMax layer is 1.
- DenseNet-121: This model aids in understanding the overfitting problem. For passing feature data, each layer has an additional information layer. Both memory productivity and computational ability are very good.
- DenseNet-201: It is a more compact model based on the idea that shorter links connecting the layers close to the input and output allow convolutional networks to be significantly deeper, better performing, and easier for training.
- InceptionV3: The image classification model InceptionV3 is commonly utilized. This network architecture has fewer parameters but more network depth. It uses the convolutional kernel splitting method to reduce the number of parameters for a faster training procedure, which is substantially faster than VGG-16.
- Xception: It has a total of 71 layers. It stands for “Extreme Inception” and can categorize photos into a thousand distinct groups of objects, such as mice, pencils, keyboards, and so on. The framework’s feature extraction base is formed by 36 convolutional layers of the Xception architecture.

### Custom CNN model

**Fig. 5:**
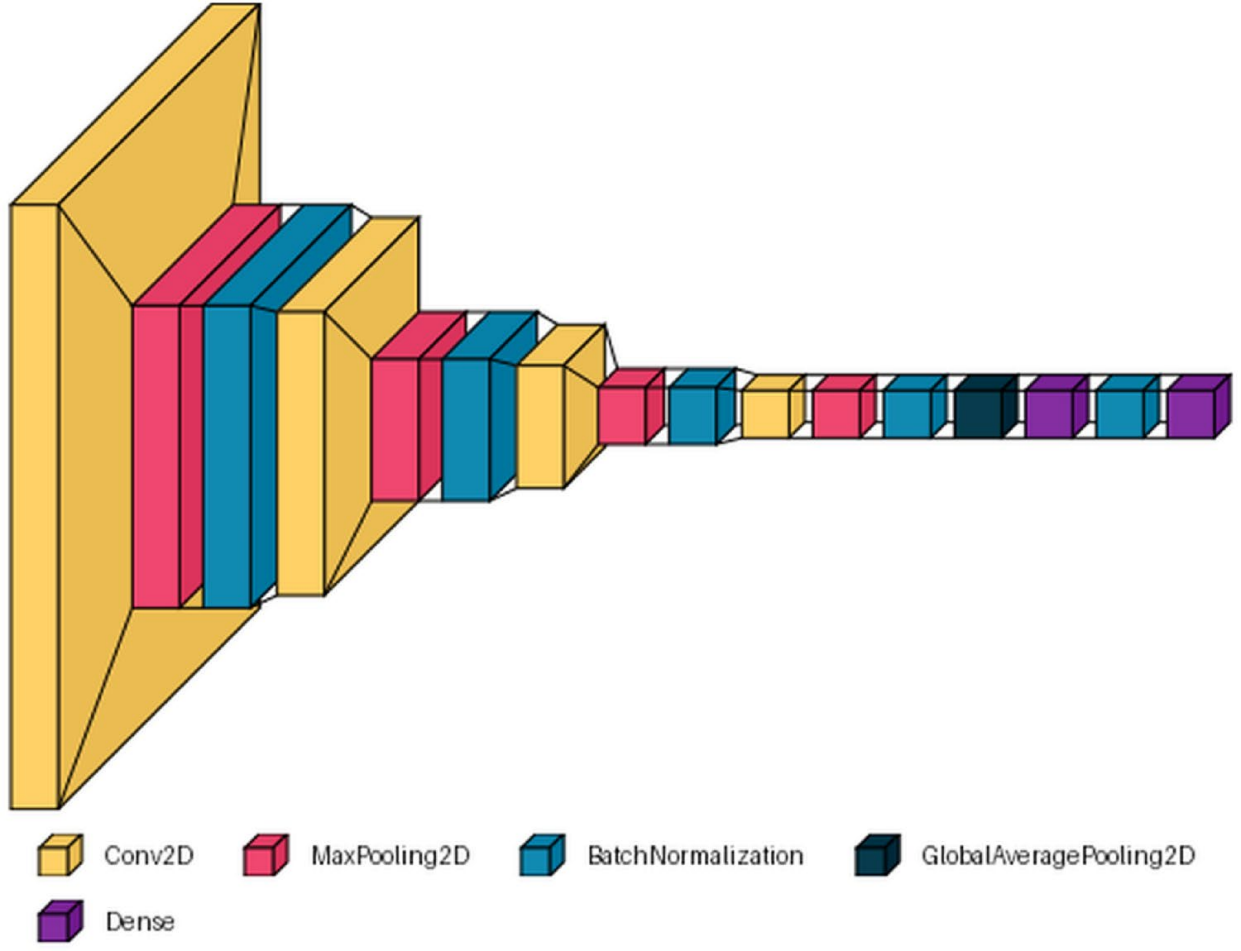
Our own custom CNN-based model architecture.

A custom CNN model (Fig. 5) was built from scratch that consisted of four convolutional blocks with Conv2D to extract features and MaxPooling2D to perform downsampling of the images. Then, BatchNormalization layer was added in between the layers to improve the model’s performance with respect to its training and validation accuracies. The feature maps must be reduced to a vector using global average pooling or GAP layer in order to make predictions. It returns a one-dimensional tensor based on an overall activation value in every characteristic map. This layer completes the model’s feature extraction section.

A dense layer would be added with 64 nodes and kernel regularizer with 0.001 training parameters after the GAP layer but before the prediction layer. This additional completely linked layer provides for higher intricacy in the interactions between the retrieved features of the convolutional blocks and predictions. A batch normalization layer will also be included to make sure that all activation values from the preceding dense layer adhere to an equivalent scale for all of the batches by converting the results into Z-scores. Finally, the output layer was added which gives the output at the end. The Dense() function has been used for the same. It takes parameter 5 for the SIPaKMeD dataset and parameter 7 for the Herlev dataset because of the number of cell types in each dataset. Also, the activation function employed is known as softmax because this is a multi-class problem. The parameters of each custom CNN layer are summarized in Table 2.

**Table 2:** Layer-wise parameters of the custom CNN architecture.

Layer type	Output shape	Kernel/pool size	Activation	Parameters
Conv2D	(64, 64, 32)	3x3	ReLU	896
MaxPooling2D	(32, 32, 32)	2x2	–	0
BatchNormalization	(32, 32, 32)	–	–	128
Conv2D	(30, 30, 32)	3x3	ReLU	9248
MaxPooling2D	(15, 15, 32)	2x2	–	0
BatchNormalization	(15, 15, 32)	–	–	128
Conv2D	(13, 13, 64)	3x3	ReLU	18,496
MaxPooling2D	(6, 6, 64)	2x2	–	0
BatchNormalization	(6, 6, 64)	–	–	256
Conv2D	(4, 4, 64)	3x3	ReLU	36,928
MaxPooling2D	(2, 2, 64)	2x2	–	0
BatchNormalization	(2, 2, 64)	–	–	256
GlobalAveragePooling2D	(64)	–	–	0
Dense	(64)	–	ReLU	4160
BatchNormalization	(64)	–	–	256
Dense (output)	(5)	–	Softmax	325
Total parameters	–	–	–	**71,077**
Trainable parameters	–	–	–	**70,565**
Non-trainable parameters	–	–	–	**512**

Significant values are in bold.

Adam optimiser is used for the custom model. The ModelCheckpoint function is utilized, which allows the most suitable model to be automatically saved to a file after the training process. As the labels of the considered dataset are categorical and not one-hot-encoded, we must choose the loss function known as categorical cross-entropy. Our own custom CNN model was trained for 20 epochs over the augmented data for both SIPaKMeD and Herlev datasets.

### Segmentation

#### Using Otsu’s thresholding

The process of separating foreground pixels from background pixels is known as thresholding. One method for achieving optimal thresholding is known as Otsu’s method, which was proposed by^34^. Otsu’s variance-based technique is used to determine the value of the threshold with the lowest weighted variation between the background and foreground pixels. It is a thresholding algorithm for global images. The important concept here is to navigate through every one of the available values of the threshold and identify the distribution of background and foreground pixels. Then, determine the criterion that has the smallest spread.

**Fig. 6:**
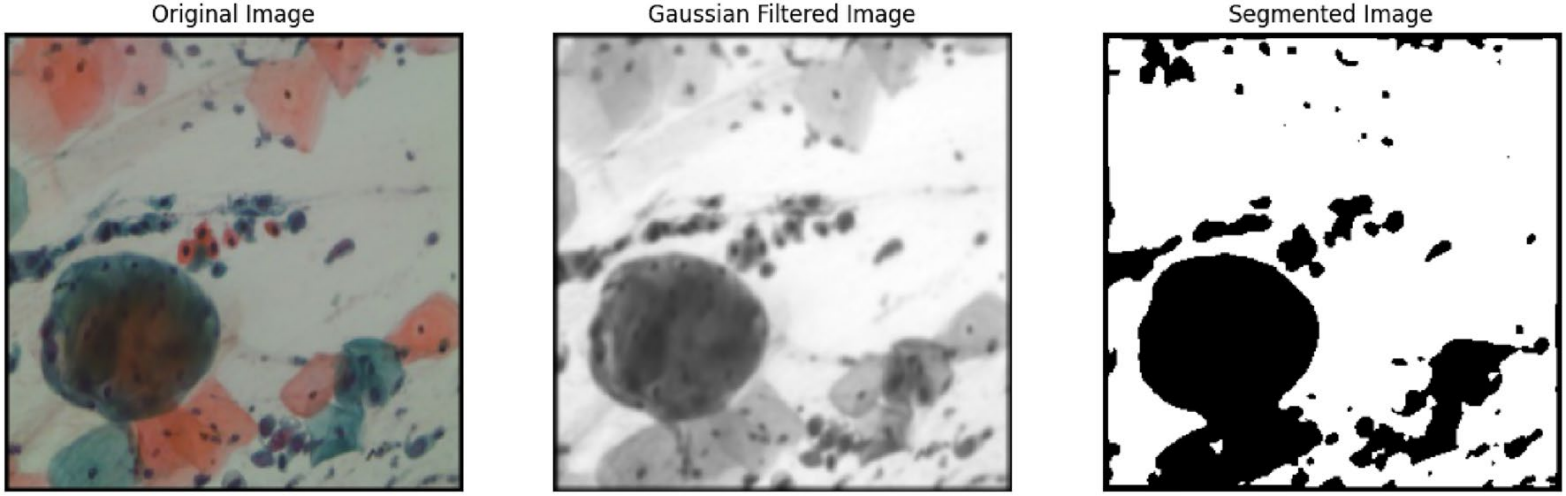
Segmentation over dyskeratotic cell cluster of SIPaKMeD WSI dataset.

We must first read the image in grayscale mode and then improve it with a Gaussian blur to reduce noise. After applying Gaussian filtering to the image, Otsu’s thresholding method is applied using OpenCV. It was first applied over a sample cell cluster from the SIPaKMeD WSI dataset as shown in Fig. 6 and later applied to all the augmented images. The method iteratively seeks the within-class variation, which is the weighted average of the variances of the two categories (background and foreground). Grayscale colours usually range between 0 and 255 is 0–1 for float. As a result, assuming we set a threshold of 100, every pixel with values less than 100 will be considered the image’s background, whereas those pixels with values that are equal to or greater than 100 would be considered the image’s foreground.

The following is the formula for computing the within-class variance at any threshold t:

5 $$\begin{aligned} \sigma ^2(t)=\omega _{bg}(t)\sigma ^2_{bg}(t)+\omega _{fg}(t)\sigma ^2_{fg}(t) \end{aligned}$$

where $\omega _{bg}(t)$ and $\omega _{fg}(t)$ represent the likelihood of the total quantity of pixels in every category at threshold t, and t and $\sigma ^2$ represents the variation in colour value.

#### Using Canny edge detection

**Fig. 7:**
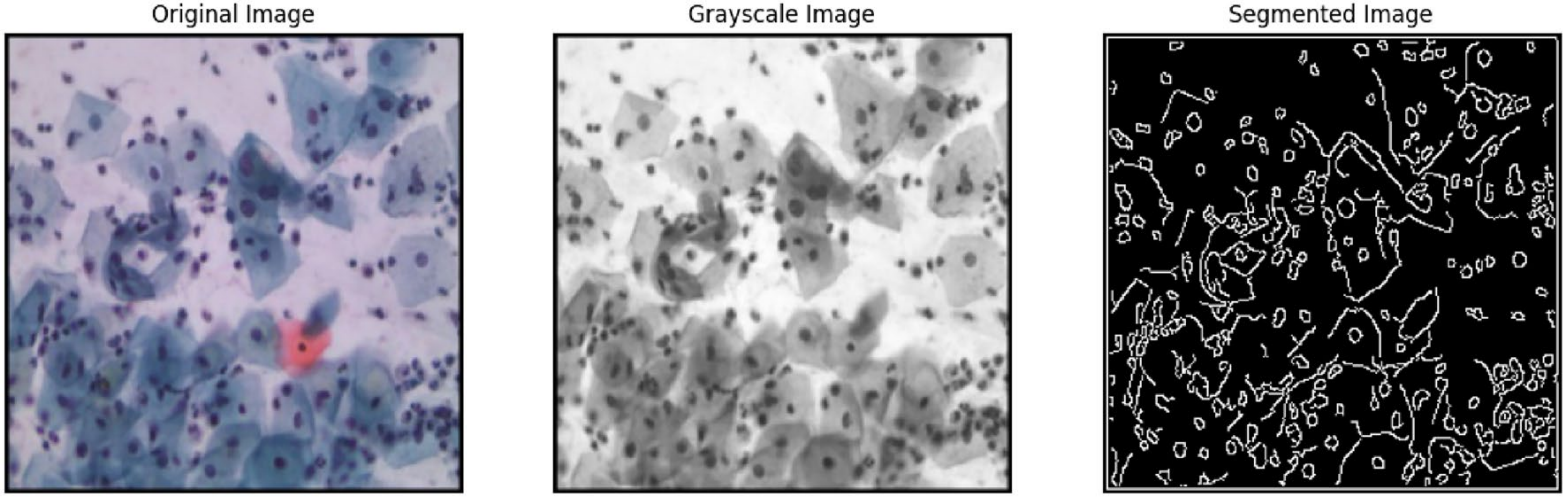
Segmentation over koilocytotic cell cluster of SIPaKMeD WSI dataset.

The Canny edge detection method employs a multi-stage approach. It is a tool for detecting different edges in images. It was initially founded in 1986 by John F. Canny. The Canny edge detection technique consists of noise reduction, non-maximum suppression, double threshold, gradient computation, and edge tracking via hysteresis stages. Following these steps, we obtain the segmented image shown in Fig. 7.

Since edge detection is susceptible to the noise of the image, the initial procedure is to use a 5x5 Gaussian filter to remove the noise. The smoothed image then undergoes filtering with a Sobel kernel in both vertical and horizontal directions in order to produce the initial derivatives-horizontal $(G_x)$ and vertical $(G_y)$. The Sobel kernels used for this operation are:

$$\begin{aligned} G_x = \begin{bmatrix} -1 & 0 & +1 \\ -2 & 0 & +2 \\ -1 & 0 & +1 \end{bmatrix},\quad G_y = \begin{bmatrix} +1 & +2 & +1 \\ 0 & 0 & 0 \\ -1 & -2 & -1 \end{bmatrix} \end{aligned}$$

Using these two images, we can calculate the edge gradient as well as the direction for every single pixel. Equation 6 represents the edge gradient and Equation 7 represents the direction for each pixel as shown in the following formula:

6 $$\begin{aligned} Edge\_Gradient (G)=\sqrt{G^2_x+G^2_y} \end{aligned}$$

7 $$\begin{aligned} Angle (\theta )=\tan ^{-1} \left( \frac{G_y}{G_x} \right) \end{aligned}$$

where $G_x$ represents the horizontal direction and $G_y$ represents the vertical direction.

After selecting the magnitude and direction of the gradient, the whole image is processed to weed out undesired pixels that may or may not be at the edge. Each individual pixel is evaluated to determine if a maximum value exists in the neighborhood of that pixel in the gradient direction.

The final phase distinguishes between genuine and incorrect edges. For this, two threshold values are required: minVal and maxVal. Such edges with a magnitude gradient more than maxVal are guaranteed to be edges, but any edges with a magnitude gradient less than minVal are guaranteed to be non-edges and should be rejected. Edges and non-edges are those having connectivity between both of these thresholds. If they are connected to “sure-edge” pixels, these are regarded as edges, otherwise they are also discarded. This stage reduces small pixel noises based on the notion that edges represent long lines. As a result, the image’s edges are now sharp. In our study, we used the OpenCV cv2.Canny() function to segment images from the SIPaKMeD WSI dataset.

#### Using region-based segmentation

**Fig. 8:**
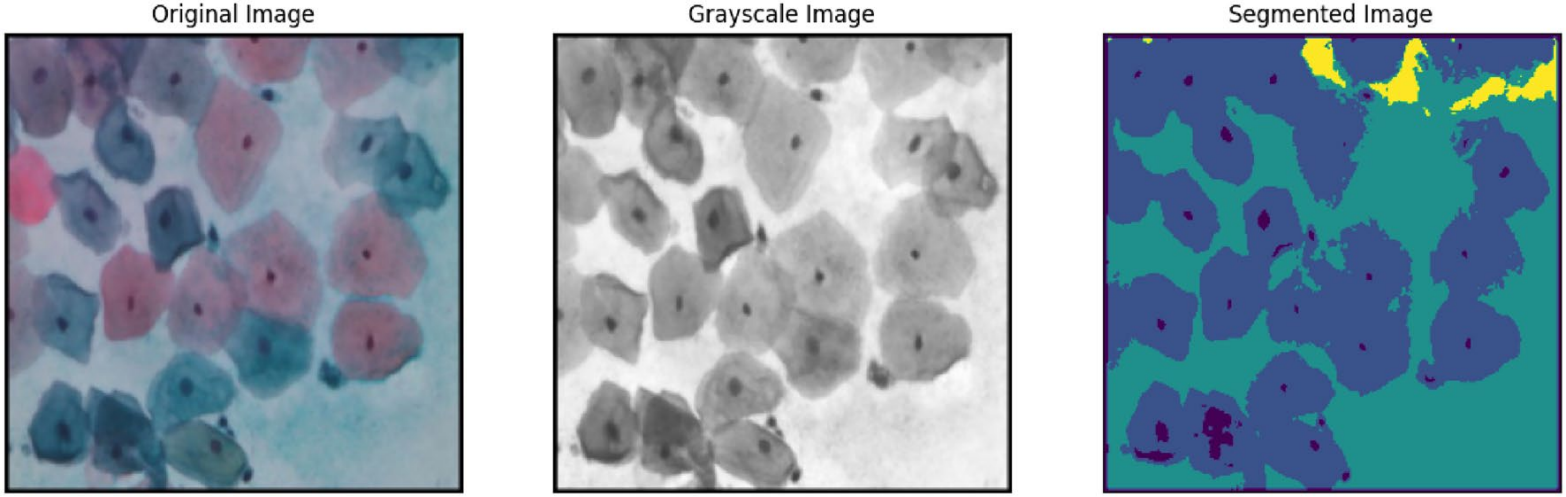
Segmentation over superficial-intermediate cell cluster of SIPaKMeD WSI dataset.

A region is described as a group of linked pixels with comparable characteristics. Pixels might be comparable in terms of brightness, colour, and other factors. To be grouped into similar pixel regions in this sort of segmentation, a pixel must adhere to a set of predetermined rules. In the case of a noisy image, region-based segmentation methods are preferable to edge-based segmentation methods. The objects are divided into different regions based on some threshold value (s). Using this method, we obtain the segmented image shown in Fig. 8.

With the Region growing method, we start with a seed pixel and inspect the neighboring pixels after that. The seed pixel is added to the area around it, and the procedure is repeated until there is no longer any similarity if the neighboring pixels adhere to the predetermined rules. The bottom-up approach is used in this method. In the event that a region expands, the preferred rule can be set as a threshold. By recursively incorporating nearby pixels that are related to and comparable to the seed pixel, we can grow regions in this segmentation. For regions with homogeneous gray levels, we use similarity measures such as gray level differences. We use connectivity to prevent different parts of the image from being connected.

### Transfer learning

Recent research efforts have primarily focused on improving the accuracy of classification tasks, particularly for large datasets using advanced methods. Traditional methods perform adequately on small datasets, but the results on large datasets are unsatisfactory. CNN has recently been used in large image datasets. In many applications, it achieves extremely high classification accuracies.

In the medical field, particularly for imaging of cervical cancer, deep learning often needs big datasets to train the networks. Transfer learning has shown to be an effective solution to deal with such issues. By first training a CNN on a domain with a lot of data, transfer learning may be accomplished. The CNN would then be retrained by fine-tuning its weights on a smaller, alternative domain. The benefits of transfer learning went beyond the problem of insufficient data, where it was shown to be a reliable initialization method for creating strong deep learning models.

We employed previously trained weights acquired after the model is trained on a large set of data, ImageNet, while retraining the CNN models on the SIPaKMeD and Herlev datasets to employ transfer learning in our study. The augmented image datasets were initially used to fine-tune the model. Using the SIPaKMeD and Herlev datasets, we employed a transfer learning technique for training the ResNet-50, VGG-16, VGG-19, DenseNet-121, DenseNet-201, InceptionV3, and Xception pre-trained CNN models for multi-class classification.

## Results

### Performance metrics

We selected a number of performance criteria that are regularly used in the literature to assess the classification performance of the proposed study for cervical cancer detection. Accuracy, precision, recall (sensitivity), and F1-score are the metrics that we measure for the problem stated in this paper.

The percentage of correctly categorized testing samples in the complete test set is referred to as accuracy. It tells you how many times the ML model was correct overall.

8 $$\begin{aligned} Accuracy = \frac{TP+TN}{TP+TN+FP+FN} \times 100\% \end{aligned}$$

where *TP* represents true positive, *TN* represents true negative, *FP* represents false positive, and *FN* represents false negative.

Precision, also called a positive predicted value, defines how good the model is at predicting a specific category. It assesses the model’s ability to correctly classify a sample as positive.

9 $$\begin{aligned} Precision = \frac{TP}{TP+FP} \times 100\% \end{aligned}$$

where *TP* represents true positive, and *FP* represents false positive.

The number of samples being tested correctly categorized as positive in every sample having the positive ground truth is referred to as recall (sensitivity) and is crucial for any medicinal application. It is extremely important for any medical application. False negatives cannot be tolerated if the models are employed in the medical area. It tells you how many times the model was able to detect a specific category.

10 $$\begin{aligned} Recall = \frac{TP}{TP+FN} \times 100\% \end{aligned}$$

where *TP* represents true positive, and *FN* represents false negative.

Although accuracy is a good performance metric to show the success of the classifier, in many health-based classification problems recall (sensitivity) becomes more important. Correct determination of cancerous cells is more vital than the correct determination of non-cancerous cells since incorrect decisions may delay the treatment time which is crucial in cancer-like diseases. Therefore, high sensitivity signals good classification performance besides accuracy.

The F1-score measures the balance between precision and recall, offering a better assessment of a model’s reliability in identifying positive cases. It is especially important in medical applications where minimizing false negatives is critical. A higher F1-score indicates stronger overall performance.

11 $$\begin{aligned} F1-score = 2 \times \frac{Precision \times Recall}{Precision + Recall} \end{aligned}$$

### Experimental results

In this paper, for the SIPaKMeD dataset, we considered the problem as a 5-class classification problem and the Herlev database as a 7-class problem related to classification and compared the performance of seven pre-trained neural networks on classifying cervical cells. The training dataset in this study was relatively small, and a deep neural network needs larger datasets. Therefore, we applied augmentation techniques in this work, and the total number of images in each class increased by eight times in the SIPaKMeD dataset and by thirty times in the Herlev dataset. The single-cell images are of size 66x66x3, and the whole slide images are of size 260x260x3. To validate the performance of our models, we followed a standard dataset split approach. Specifically, the available data was partitioned into three non-overlapping subsets: 80% of the images were used for training, 10% for validation, and the remaining 10% for testing. The validation set was used to fine-tune hyperparameters and monitor training performance to avoid overfitting, while the final performance metrics were reported on the held-out test set, which remained unseen throughout training.

The experiments were performed in three stages for the single-cell image datasets. In the first stage, the original images of the SIPaKMeD FCI (full cell images) and Herlev datasets were given to the previously stated deep CNN models. That is, no preprocessing was performed. The performance metrics obtained for each pre-trained network are shown in Table 3 for the SIPaKMeD FCI dataset, and Table 4 for the Herlev dataset. The best accuracy belongs to the classification using DenseNet201 and DenseNet121 for the SIPaKMeD FCI dataset and Herlev dataset respectively. The best recall (sensitivity) is obtained by DenseNet121 for each of the SIPaKMeD FCI as well as the Herlev datasets.

**Table 3:** Performance metrics over the original images of SIPaKMeD FCI dataset.

Models	Accuracy (in %)	Precision (in %)	Recall (in %)	F1-score (in %)
ResNet50	47.06	75.67	6.86	12.57
VGG16	81.37	87.53	74.02	80.17
VGG19	75.49	84.36	70.09	76.57
DenseNet121	85.05	86.83	84.07	85.42
DenseNet201	85.54	86.80	83.82	85.28
InceptionV3	76.96	78.61	72.06	75.18
Xception	82.84	83.84	81.37	82.59

**Table 4:** Performance metrics over the original images of Herlev dataset.

Models	Accuracy (in %)	Precision (in %)	Recall (in %)	F1-score (in %)
ResNet50	25.53	0	0	0
VGG16	48.93	69.23	9.57	16.82
VGG19	45.74	81.81	9.57	17.14
DenseNet121	54.25	60	47.87	53.25
DenseNet201	48.93	57.14	42.55	48.78
InceptionV3	50	72.97	28.72	41.22
Xception	52.13	58.46	40.42	47.79

In the second stage, the augmentation technique is applied to the data, and the pre-trained models are applied to the newly augmented image data. The newly added images were obtained by rescaling, shifting, and rotating operations in vertical and horizontal directions, zooming and blurring. Tables 5 and 6 illustrate the performance metrics achieved for each pre-trained network. The classification using Xception has the best accuracy, recall, precision, and F1-score for both the SIPaKMeD FCI and Herlev datasets. On comparing the results of the original data, we can see that the augmentation technique helps to increase the accuracy of the models.

**Table 5:** Performance metrics over the augmented images of SIPaKMeD FCI dataset.

Models	Accuracy (in %)	Precision (in %)	Recall (in %)	F1-score (in %)
ResNet50	68.11	81.08	49.07	61.14
VGG16	82.59	87.26	77.51	82.10
VGG19	80.46	86.13	74.94	80.15
DenseNet121	88.06	89.42	86.79	88.09
DenseNet201	87.28	89.06	85.33	87.16
InceptionV3	87.03	87.98	86.26	87.11
Xception	95.01	95.49	94.48	94.98

**Table 6:** Performance metrics over the augmented images of Herlev dataset.

Models	Accuracy (in %)	Precision (in %)	Recall (in %)	F1-score (in %)
ResNet50	45.47	68.19	20.89	31.98
VGG16	52.57	77.09	26.30	39.22
VGG19	49.36	72.51	25.70	37.95
DenseNet121	58.18	70.57	44.71	54.74
DenseNet201	53.33	72.99	34.68	47.02
InceptionV3	52.45	73.74	32.32	44.94
Xception	89.77	90.61	88.65	89.62

**Fig. 9:**
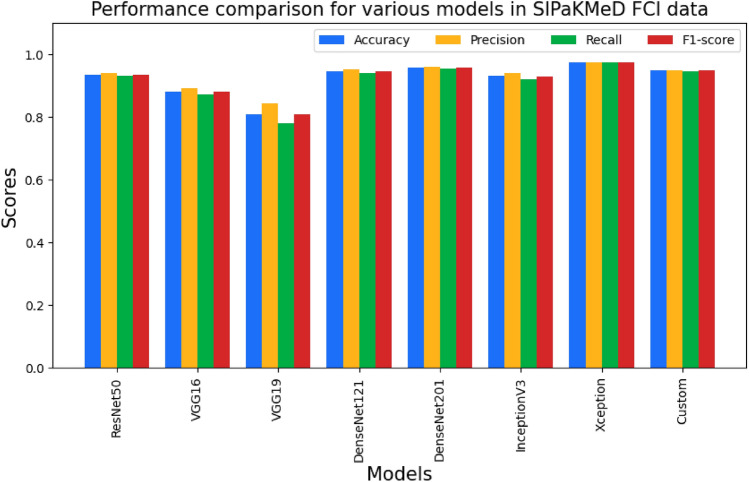
Graph showing the performance comparison of various CNN models for SIPaKMeD FCI dataset.

**Fig. 10:**
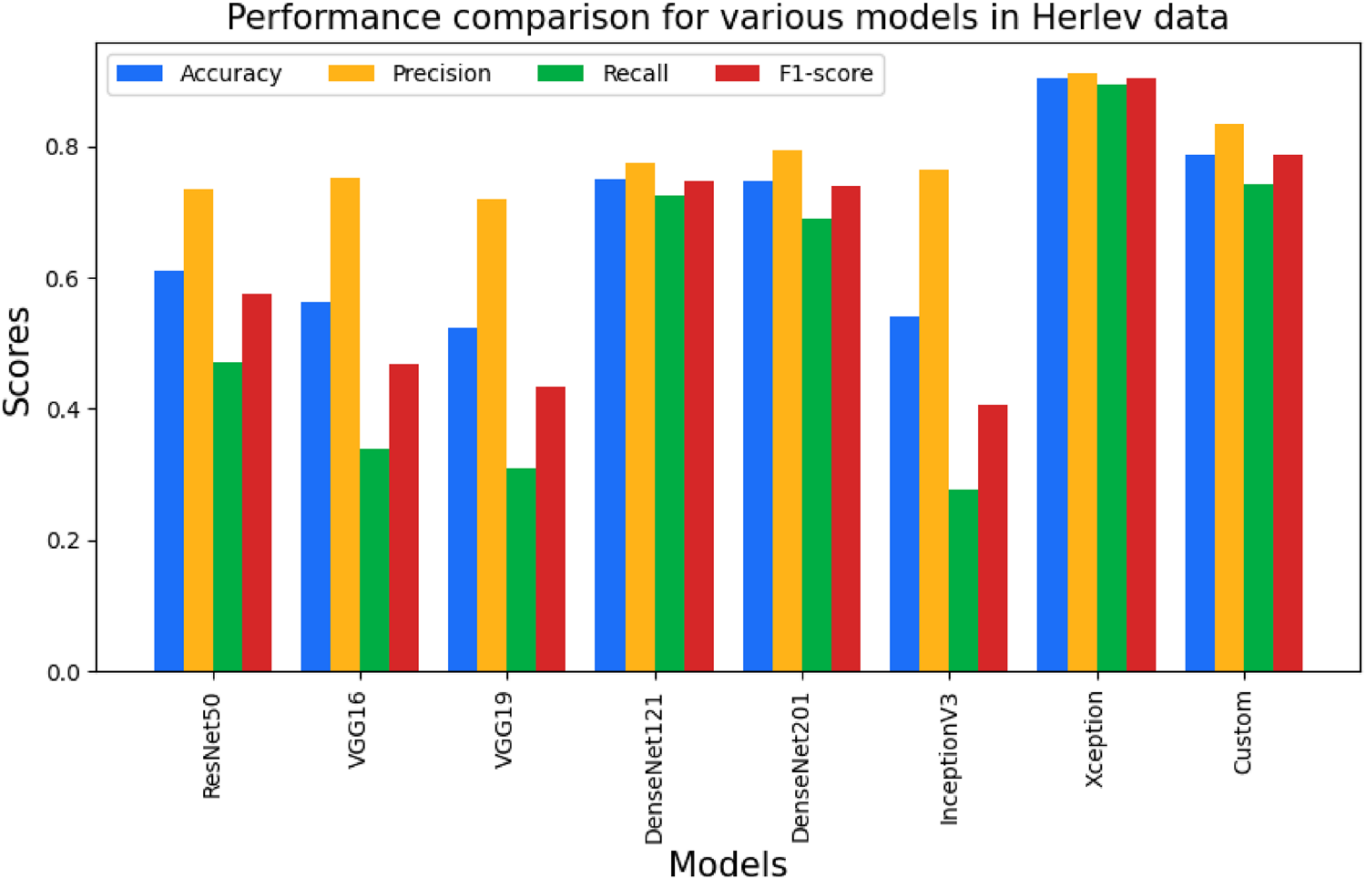
Graph showing the performance comparison of various CNN models for Herlev dataset.

The third stage was also with the augmented data, but we intended to demonstrate the influence of the L2 Regularization parameter and Batch Normalization approach. To reduce overfitting, the regularization parameter is utilized. The L2 Regularization parameter is set to 0.001. Then batch normalization was introduced in between the neural layers of the deep models. As a result of this parameter adjustment, the performances of all network models improved. The performance metrics obtained for each pre-trained network and our custom CNN model are shown in Tables 7 and 8. The best performance is shown by the classification using Xception for cervical cell classification for both the SIPaKMeD FCI and Herlev datasets. Figures 9 and 10 show the performance comparison in terms of precision, recall, F1-score, and accuracy pertaining to the different CNN models for the SIPaKMeD FCI and Herlev datasets.

**Table 7:** Performance metrics over the augmented images (with regularization and normalization) of SIPaKMeD FCI dataset.

Models	Accuracy (in %)	Precision (in %)	Recall (in %)	F1-score (in %)
ResNet50	93.58	94.13	93.11	93.62
VGG16	88.12	89.25	87.09	88.16
VGG19	80.82	84.30	77.97	81.01
DenseNet121	94.66	95.23	94.19	94.71
DenseNet201	95.81	96.19	95.56	95.87
InceptionV3	93.21	94.06	91.96	93
Xception	97.55	97.58	97.55	97.56
Custom	94.79	95.04	94.57	94.80

**Table 8:** Performance metrics over the augmented images (with regularization and normalization) of Herlev dataset.

Models	Accuracy (in %)	Precision (in %)	Recall (in %)	F1-score (in %)
ResNet50	61.06	73.41	47.15	57.42
VGG16	56.25	75.27	33.92	46.77
VGG19	52.33	72.03	30.87	43.22
DenseNet121	74.82	77.33	72.33	74.75
DenseNet201	74.74	79.46	69.01	73.87
InceptionV3	54.01	76.33	27.67	40.62
Xception	90.42	91.19	89.25	90.21
Custom	78.75	83.42	74.26	78.57

**Fig. 11:**
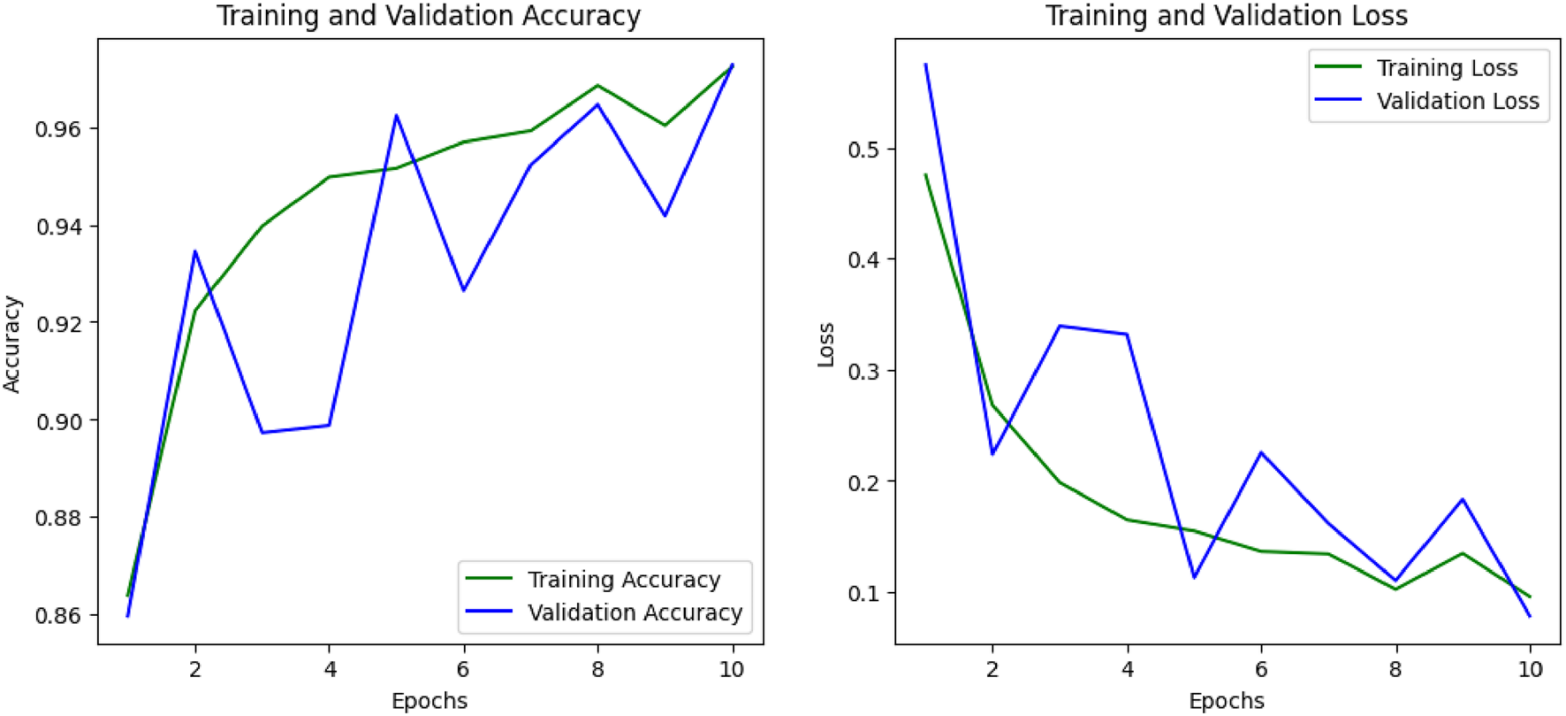
Training and validation graphs for the Xception-based model’s accuracy and loss on the SIPaKMeD FCI dataset.

**Fig. 12:**
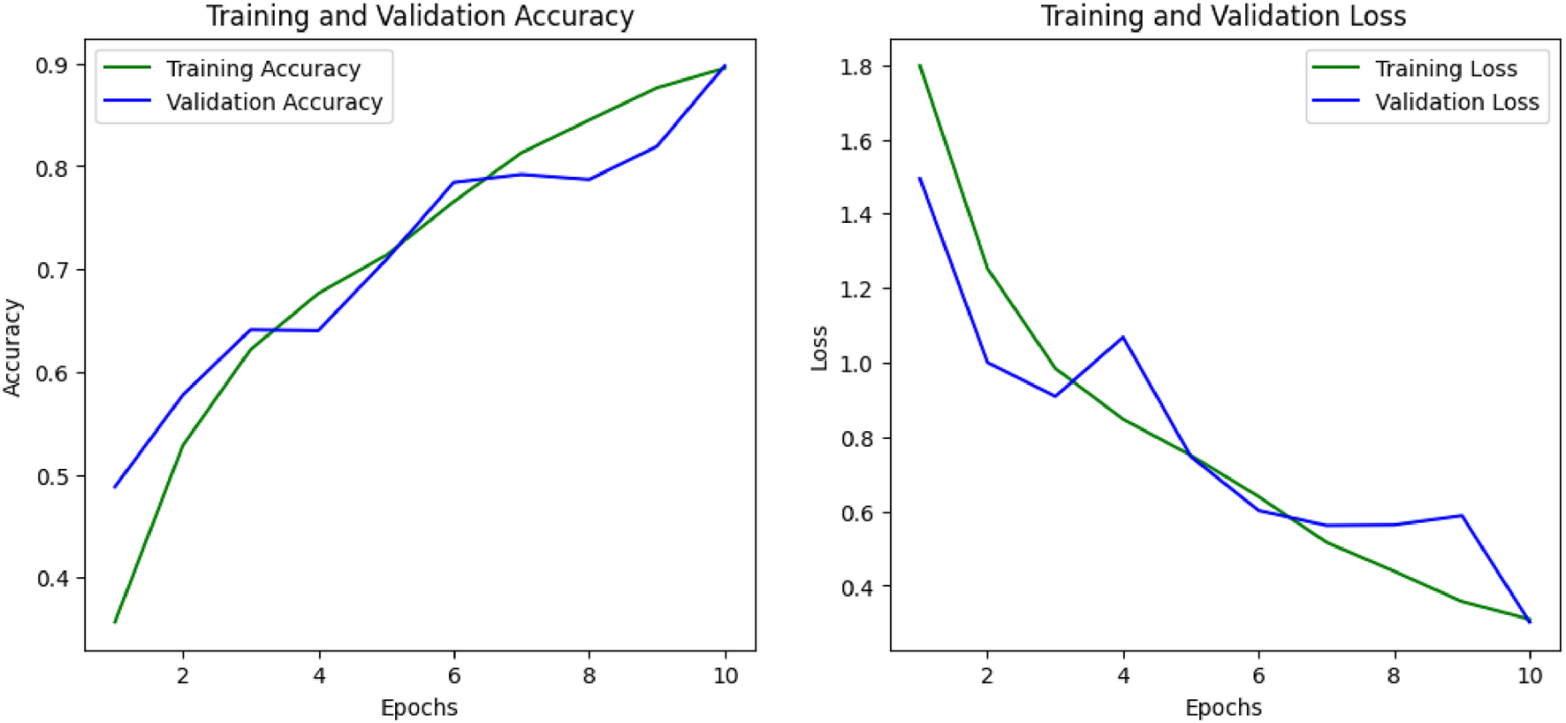
Training and validation graphs for the Xception-based model’s accuracy and loss on the Herlev dataset.

Figures 11 and 12 demonstrate the training as well as validation accuracy along with loss for the CNN model that outperforms all eight models for the SIPaKMeD FCI and Herlev datasets respectively. Here, for both these datasets, the Xception-based deep CNN model gives the highest accuracy when applied over the augmented data with regularization and normalization methods. From the graphs, we can see where the loss and accuracy occurred between the model training and validation. The performance of the model is good since the number of nodes in the hidden layers has increased which means that the algorithm learns better and fits more properly. The reduction in the gap between training and validation performance, along with improved evaluation metrics, confirms that the use of regularization and batch normalization effectively mitigated overfitting in our experiments.

Now, unlike the previous cell images of the SIPaKMeD FCI and Herlev datasets, the SIPaKMeD WSI dataset consists of cluster cell images, where we worked on whole slide Pap smear test images. For this dataset, we first performed a direct categorization of cervical clusters of cells without segmentation and refrain from extracting the individual cells. Similar to the previous experiments mentioned above, the pre-trained CNN models are first applied over the original images of the SIPaKMeD WSI dataset and the performance is observed as shown in Table 9. The DenseNet201 model gave the best accuracy, recall, and F1-score.

**Table 9:** Performance metrics over the original images of SIPaKMeD WSI dataset.

Models	Accuracy (in %)	Precision (in %)	Recall (in %)	F1-score (in %)
ResNet50	29.29	29.29	29.29	29.29
VGG16	75.76	78.89	71.72	75.13
VGG19	58.59	63.33	57.58	60.32
DenseNet121	77.78	77.78	77.78	77.78
DenseNet201	78.79	78.79	78.79	78.79
InceptionV3	53.53	53.61	52.52	53.06
Xception	61.62	62.24	61.62	61.93

Now again augmentation techniques are adopted with the same parameters over the whole slide images. Then, the pre-trained models are applied over the augmented data and the performance metrics obtained are shown in Table 10. The Xception-based deep learning approach gave the best performance over the augmented data.

**Table 10:** Performance metrics over the augmented images of SIPaKMeD WSI dataset.

Models	Accuracy (in %)	Precision (in %)	Recall (in %)	F1-score (in %)
ResNet50	41.15	41.17	39.45	40.29
VGG16	78.49	79.49	78.36	78.92
VGG19	77.18	77.82	76.40	77.10
DenseNet121	78.09	78.30	78.09	78.19
DenseNet201	80.96	80.96	80.96	80.96
InceptionV3	59.06	76.41	29.99	43.07
Xception	86.44	89.13	84.48	86.74

**Fig. 13:**
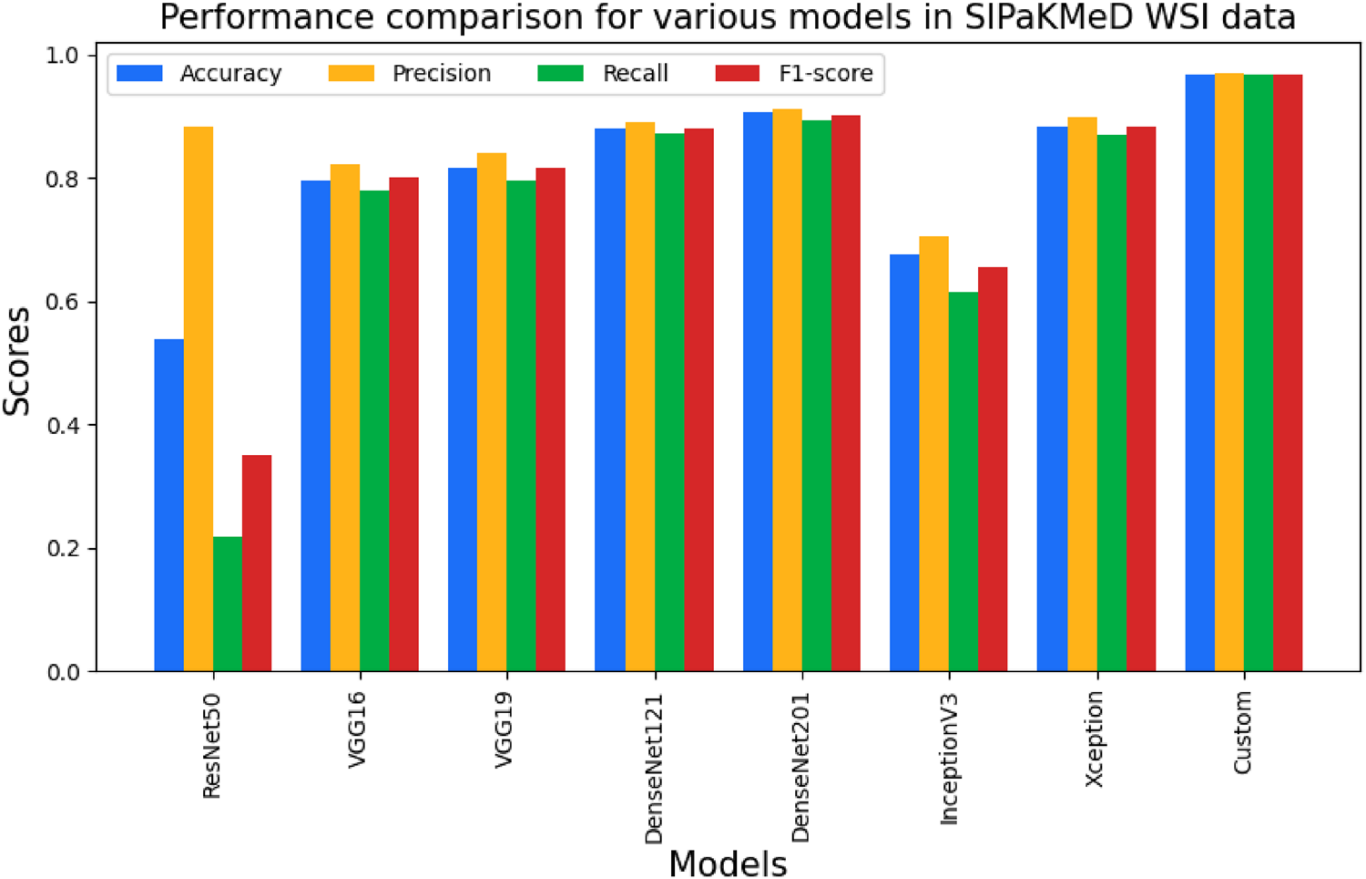
Graph showing the performance comparison of various CNN models for SIPaKMeD WSI dataset.

Then regularization and normalization strategies were used over the augmented data and the pre-trained models and our custom CNN model are applied, and performance is observed as shown in Table 11. Our own custom CNN-based model outperformed other models with respect to precision, recall, F1-score, and accuracy. Figure 13 depicts the performance comparison of the different CNN models for the SIPaKMeD WSI dataset.

**Table 11:** Performance metrics over the augmented images (with regularization and normalization) of SIPaKMeD WSI dataset.

Models	Accuracy (in %)	Precision (in %)	Recall (in %)	F1-score (in %)
ResNet50	53.85	88.36	21.77	34.93
VGG16	79.40	82.23	77.84	79.97
VGG19	81.75	83.91	79.53	81.66
DenseNet121	88.01	88.95	87.09	88.01
DenseNet201	90.61	91.09	89.31	90.19
InceptionV3	67.54	70.40	61.41	65.60
Xception	88.14	89.77	86.96	88.34
Custom	96.74	97.12	96.61	96.86

We then applied a few segmentation strategies over the whole slide images and then applied the CNN models with transfer learning over the segmented images. For the SIPaKMeD WSI dataset, we first applied Otsu’s thresholding method for segmenting the cell patches. After performing segmentation, the CNN models that were previously trained are applied to utilize transfer-learning methods, and performance metrics obtained for the various models are shown in Table 12. The Xception model shows the highest performance with 52.28% accuracy and 63.16% precision, which is low when compared with the results in Table 11.

**Table 12:** Performance metrics over the segmented images (Otsu’s thresholding) with transfer learning of SIPaKMeD WSI dataset.

Models	Accuracy (in %)	Precision (in %)	Recall (in %)	F1-score (in %)
ResNet50	38.85	63.01	5.99	10.94
VGG16	48.37	58.59	27.12	37.08
VGG19	44.72	57.56	20.34	30.06
DenseNet121	45.76	57.43	25.68	35.49
DenseNet201	50.06	58.84	32.98	42.27
InceptionV3	28.16	0	0	0
Xception	52.28	63.16	29.73	40.43
Custom	40.28	51.53	13.17	20.98

Then the Canny edge detection method was applied over the whole slide images and then again the CNN models were applied with transfer learning. The model performance is shown in Table 13. The Xception model shows the highest performance with 58.93% accuracy, 51.63% recall, and 56.90% F1-score. The results obtained here are still low when compared with that in Table 11.

**Table 13:** Performance metrics over the segmented images (Canny edge detection) with transfer learning of SIPaKMeD WSI dataset.

Models	Accuracy (in %)	Precision (in %)	Recall (in %)	F1-score (in %)
ResNet50	43.02	60.28	22.16	32.41
VGG16	53.19	64.71	37.29	47.31
VGG19	52.41	63.15	35.07	45.10
DenseNet121	55.80	64.67	40.81	50.04
DenseNet201	54.63	60.46	44.85	51.50
InceptionV3	33.38	80.36	5.87	10.94
Xception	58.93	63.36	51.63	56.90
Custom	53.72	60.35	40.68	48.60

Finally, the region-based segmentation method was applied with 5 folds. After segmenting the images, the various CNN models are applied and their accuracy, precision, recall, and F1-score were observed as shown in Table 14. Here, the Xception model with transfer learning shows an accuracy of 70.18% and a recall score of 67.45%, which again is low. When the custom CNN model was applied, it gave improved results compared to the previously trained deep CNN models when transfer learning techniques were used.

**Table 14:** Performance metrics over the segmented images (region-based) with transfer learning of SIPaKMeD WSI dataset.

Models	Accuracy (in %)	Precision (in %)	Recall (in %)	F1-score (in %)
ResNet50	34.64	59.09	5.08	9.36
VGG16	60.03	66.21	50.78	57.48
VGG19	56.77	66.46	41.79	51.31
DenseNet121	64.32	71.67	54.69	62.04
DenseNet201	66.28	71.18	58.20	64.04
InceptionV3	28.26	0	0	0
Xception	70.18	73.79	67.45	70.48
Custom	72.39	75.78	69.27	72.38

## Discussion

In this study, we put forward a strategy for detecting and analysing cervical cancer proliferation using medical pap smear test images. To train the neural network model and survey its assessment, we use the deep convolutional neural network model and the SIPaKMeD and Herlev datasets. The evaluation is carried out by considering various metrics such as accuracy, recall, precision, and F1-score.

The results of the original unprocessed datasets, as shown in Tables 3, 4, and 9, consistently showed suboptimal performance due to significant class imbalance and limited sample sizes, particularly in underrepresented categories. As a result, the study shifted focus to evaluate model performance with data augmentation and regularization techniques applied to the image dataset. The performance improved significantly compared to the raw images, as reflected in Tables 5, 6, and 10. Regularization and normalization strategies were introduced to reduce overfitting, leading to further performance improvements, as shown in Tables 7, 8, and 11. Due to these improvements, direct comparisons with the results of the raw dataset were excluded to avoid misleading interpretations and to emphasize the practical advantages of using augmentation in biomedical image classification tasks.

The accuracy values obtained by the Xception-based deep learning approach with regularization and normalization for the early diagnosis of cervical cancer are remarkable for the SIPaKMeD cell dataset as shown in Table 7. Its other metrics also show the success of the proposed method. Similarly for the Herlev cell dataset, the Xception model gave the highest performance, followed by our own custom CNN-based model as shown in Table 8.

**Fig. 14:**
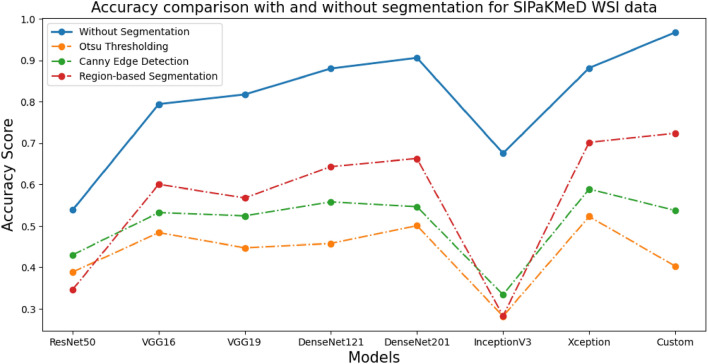
Graph showing the accuracy comparison with and without segmentation for SIPaKMeD WSI dataset.

For the SIPaKMeD WSI data, direct categorization of cervical cell groups without any segmentation was performed, and our own custom CNN model showed the best performance. The results shown in Table 11 also indicate that there is no obstacle caused by overlapped cells given that we identify the WSI patches explicitly devoid of cropping. From Tables 12, 13, and 14, we see that even after trying out three different segmentation strategies, the results did not improve from before. This is probably because whole slide images are the most difficult to segment. On comparing the results with and without segmentation as clearly depicted in Fig. 14, the latter is better for this dataset. Hence we can interpret that the segmentation process or the extraction of cell patches is not exactly necessary for the models to give a good performance.

This study presents a robust deep learning framework for cervical cell classification using the SIPaKMeD and Herlev datasets. To demonstrate the efficacy of the suggested method, we conducted a detailed comparison with several existing methods from the literature. We compared our approach over the SIPaKMeD FCI dataset, to five other existing techniques^10,31,32,35,36^ that use deep learning. Table 15 summarizes the findings, where we compared the strategies based on the accuracy values obtained. According to the table, our Xception-based model has an accuracy of 97.55% and surpasses the other five techniques (96.90%, 95.35%, 87.32%, 94.09%, and 93.70%). Because of the Xception model’s design technique, precise outcomes were obtained with a significantly lower number of features. Moreover, unlike the study in  ^30^, which primarily benchmarks CNNs without introducing methodological innovations, our study combines model exploration, segmentation analysis, and extensive tuning, providing a more practical and comprehensive approach.

**Table 15:** Comparative analysis of our proposed approach to previous works for SIPaKMeD FCI dataset.

Author	Year	Method	Accuracy (in %)
Şentürk & Süleyman	2022	SqueezeNet-based	96.90
Plissiti et al.	2018	VGG19-based	95.35
Haryanto et al.	2020	AlexNet-based	87.32
Win et al.	2020	Bagging ensemble classifier	94.09
Deo et al.	2024	CerviFormer: Transformer-based	93.70
Current study with Xception-based model	2023	Xception-based	97.55

Table 16 clearly illustrates how the suggested research methodology was able to attain improved accuracy without the expense of selecting certain characteristics or applying segmentation over the image by comparing the accuracy values obtained with that of other studies. It compares the accuracies of the proposed method, and the recent studies^25–29,33,37,38^ that use the Herlev dataset with seven-class accuracy. Our method outperforms the present approaches in the literature with an accuracy of 90.42%. The results of this study indicate that the suggested model has a couple of benefits when it comes to representation power, that results in a greater categorization accuracy. These comparative results strongly support the robustness and generalisability of the proposed approach across datasets and classification complexities.

**Table 16:** Comparative analysis of our proposed approach to previous works for Herlev dataset.

Author	Year	Method	Accuracy (in %)
Jantzen et al.	2005	Benchmark	61.1
Lin et al.	2019	Morphology & fine-grained CNN	64.5
Chen et al.	2020	Inception-ResNet+snapshot ensemble	65.56
Malli & Nandyal	2017	k-NNs and ANNs	k-NNs: 88; ANNs: 54
Promworn et al.	2019	DenseNet161	68.54
Dounias et al.	2006	C-means / Fuzzy clustering	72–80
Sarwar et al.	2015	Hybrid ensemble	78
Rahaman et al.	2021	Hybrid deep feature fusion technique	90.32
Current study with Xception-based model	2023	Xception-based	90.42

Our approach has a few limitations, despite its high performance. To begin with, in spite of the great accuracy measure of the SIPaKMeD dataset, our method performs inadequately on the Herlev dataset for the 7-class problem. A good detection method should catch all aberrant cells. To circumvent this for the multiclass classification task, we may have included segmented cell characteristics in our approach. Second, while we examined seven deep learning algorithms, adjusted the parameters according to the problem, and compared how well they performed with our proposed technique, future studies could explore other state-of-the-art architectures such as Vision Transformers (ViTs) and Recurrent Neural Networks (RNNs), which have shown promise in recent medical imaging research and could serve as strong baselines for comparison. Third, in the case of whole slide images, our suggested approach could be adapted for overlapping cell categorization. When we applied the segmentation methods to the WSI data, the results did not improve. This is probably because whole slide images are the most difficult to segment when compared to single-cell images. Hence, by applying advanced segmentation strategies like Mask-RCNN for precisely extracting single-cell patches from whole slide images, we could improve the model’s overall performance. Finally, although accuracy, precision, recall, and F1-score were included for performance evaluation, future studies could incorporate the Matthews Correlation Coefficient (MCC) to better assess model robustness under class imbalance.

The proposed CNN-based system could be integrated into clinical workflows as a decision support tool to assist pathologists in the screening of Pap smear images. Once digitized, the model can analyse the slides to identify abnormal cells for review, potentially increasing the speed and accuracy of the diagnostic. Future deployment would require external validation, user interface development, and integration with hospital information systems to support real-time use without replacing expert oversight.

## Conclusions

In this paper, we propose a methodology where we make use of deep CNN models for detecting cervical carcinoma utilizing pap smear test images with cells categorized as healthy or abnormal. The key contribution made by this research is that we built our own custom CNN model from scratch and it has proved to give remarkable outcomes and even outperform other pre-trained models in some cases. Despite previous approaches that depend upon cytoplasm and nucleus segmentation and custom-built characteristics, our suggested method provides a complete categorization of cervical cancer cells utilizing deep characteristics. To assess the efficacy of our suggested approach, we use the SIPaKMeD and Herlev datasets. Further, we have performed a direct segmentation-free classification of the whole slide images using CNNs without any extraction of the cell patches, and that has shown promising results with the highest accuracy of 96.74% using our own custom CNN-based model.

For the 5-class classification problem, we attained an accuracy of 97.55% using the SIPaKMeD FCI dataset. We obtained an accuracy score of 90.42% on the 7-class classification problem using the Herlev dataset. The suggested methodology prevails over cutting-edge techniques for cervical cancer detection achieving high accuracy, precision, recall, and F1-score. We were able to identify that by applying data augmentation techniques and further incorporating batch normalization and regularization functions, the overall performance of the neural networks improved greatly and shows a positive impact. In addition, we avoid the drawbacks of earlier studies by (1) directly classifying the WSI patches devoid of cropping (2) avoiding bottlenecks caused by overlapping cells. We were able to infer that even without segmentation the results were good. Finally, the overall performance shows that the proposed method can be reliably and efficiently used for the classification of cervical cancerous cells from non-cancerous cells. Systems that use deep learning are capable of performing complex medical tasks involving classification such as cervical cancer screening. As a result, systems like this may be useful to healthcare professionals in determining the cause of this disease. As a result, it is critical that government agencies, medical care, and social organisations promote and encourage early cervical cancer treatment. Developing a strategy for a treatment plan customized to each patient’s individual cancer type can significantly reduce the incidence and death rate from this disease.

## Data Availability

The datasets used and/or analysed during the current study-including the Herlev dataset, SIPaKMeD-FCI, and SIPaKMeD-WSI-are publicly available in https://www.kaggle.com/datasets/bornarado/papsmeardatasets (last accessed: April 23, 2023).
